# Fecal virome composition of migratory wild duck species

**DOI:** 10.1371/journal.pone.0206970

**Published:** 2018-11-21

**Authors:** Luis Alfonso Ramírez-Martínez, Elizabeth Loza-Rubio, Juan Mosqueda, Manuel Leonardo González-Garay, Gary García-Espinosa

**Affiliations:** 1 Departamento de Medicina y Zootecnia de Aves, Facultad de Medicina Veterinaria y Zootecnia, Universidad Nacional Autónoma de México, Ciudad de México, México; 2 Departamento de Biotecnología en Salud Animal, Centro Nacional de Investigación Disciplinaria en Microbiología Animal, Instituto Nacional de Investigaciones Forestales, Agrícolas y Pecuarias, (CENID-Microbiología-INIFAP), Ciudad de México, México; 3 Facultad de Ciencias Naturales, Universidad Autónoma de Querétaro, Querétaro, Querétaro, México; 4 Department of Medicine, Center for Biomedical Informatics & Biostatistics, The University of Arizona, Tucson, Arizona, United States of America; Defence Research Laboratory, INDIA

## Abstract

The fecal virome comprises a complex diversity of eukaryotic viruses, phages and viruses that infect the host. However, little is known about the intestinal community of viruses that is present in wild waterfowl, and the structure of this community in wild ducks has not yet been studied. The fecal virome compositions of six species of wild dabbling ducks and one species of wild diving duck were thus analyzed. Fecal samples were collected directly from the rectums of 60 ducks donated by hunters. DNA and RNA virus particles were purified and sequenced using the MiSeq Illumina platform. The reads obtained from the sequencing were analyzed and compared with sequences in the GenBank database. Viral-related sequences from the *Herpesviridae*, *Alloherpesviridae*, *Adenoviridae*, *Retroviridae* and *Myoviridae* viral families showed the highest overall abundances in the samples. The virome analysis identified viruses that had not been found in wild duck feces and revealed distinct virome profiles between different species and between samples of the same species. This study increases our understanding of viruses in wild ducks as possible viral reservoirs and provides a basis for further studying and monitoring the transmission of viruses from wild animals to humans and disease outbreaks in domestic animals.

## Introduction

The microbiome in vertebrates consists of multiple microorganisms that include bacteria, fungi, archaea and viruses. The bacterial community has been widely studied, and the results have revealed that this community contributes to the health status of the host and establishes a symbiotic or commensalistic relationship with the host [1, 2]. The virome, which forms part of the microbiome, is the viral component that includes eukaryotic viruses, bacteriophages, viruses that infect host organisms and genetic elements of the virus in the host genome [3]. This viral component includes pathogenic viruses implicated in host diseases, and recent years have seen increased interest in the interactions of this component with the host and other elements of the microbiome, persistent viruses and the effects on immunomodulation and susceptibility to diseases [4–6]. Moreover, a specific-host virome profile has been suggested, but this profile can be modified by endogenous and exogenous factors [7]. In particular, factors such as age, drugs, diet and infections could have effects on the virome composition, as has been observed in chickens, pigs and humans [8–10]. However, the structure of the fecal virome in wild ducks has not been researched.

Ducks are the most abundant birds of the *Anatidae* family, and more than 20 species arrive every year at the high plateau, Pacific coast and wetlands of the Gulf of Mexico during the autumn-winter season [11]. Ducks can be generally divided into dabbling and diving ducks based on their feeding behavior in water, and different species of resident or migratory ducks commonly share habitats and nests [12]. In addition, migratory ducks play an important role as reservoirs and disseminators of *Influenzavirus* A and *Avulavirus* [13], and avian influenza viruses (H5 and H7) are occasionally transmitted from wild aquatic birds to domestic poultry and humans [14]. Additionally, differences in susceptibility to these viruses between species of wild ducks have been detected [15, 16], and the viral diversity in the feces of wild migratory ducks and the viruses carried by these birds from the aquatic ecosystem have not been fully described. Moreover, whether the virome structures in the gut of different species of wild ducks are similar or varied requires further analysis. The study of viruses in natural habitats of wild populations could provide a better understanding of the outbreaks of new infectious diseases in domestic animals and humans. Thus, the objective of this study was to characterize the virome compositions of six different species of wild dabbling ducks and one species of diving duck during their wintering stayover in a natural wetland to elucidate the viruses harbored by these birds.

## Materials and methods

### Wetland location

This study was conducted in the northern wetland at the Ciénegas of Lerma in the central high plateau of Mexico. This area is listed as a RAMSAR site of International Importance [17]. The wetland is located in the State of Mexico within the Municipality of Lerma (19°21'21.8"N, 99°31'00.5"W) and is a stopover site for migratory wild ducks from North America during the autumn and winter seasons [18]. The area is surrounded by rural areas that are used for agricultural purposes and backyard livestock, such as cattle, poultry, and swine [19].

### Sample collection

Sixty carcasses of clinically healthy wild ducks were donated by hunters during January and February of 2016. These ducks belonged to the following species: blue-winged teal (*Spatula discors*), northern pintail (*Anas acuta*), cinnamon teal (*Spatula cyanoptera*), American wigeon (*Mareca americana*), northern shoveler (*Spatula clypeata*), gadwall (*Mareca strepera*) and ruddy duck (*Oxyura jamaicensis*). The feces were obtained directly from the rectum, immediately placed in individual sterile tubes and subsequently maintained at 4°C until their transport to the laboratory for storage at -75°C. The sample collection in the present study was performed with the ethical approval of the Committee for Animal Experiments at the Faculty of Veterinary Medicine and Animal Husbandry (SICUAE, number DC-2016/2-3).

### Purification of viral particles (virus-like particles)

Individual fecal samples were diluted 1:10 (w/v) with sterile phosphate-buffered saline (PBS) and homogenized by vortexing. From the 60 stool samples, pools of five samples were prepared according to the species of duck by transfering approximately 5 ml of each of the five individual and diluted samples into a new plastic tube. The 12 pools obtained were processed according to the methods described by Day *et al*. [20] with some modifications. The pools were centrifuged at 7000 × g (Heraeus Biofuge Primo R) and 4°C for 15 minutes to clarify the contents. The supernatants were collected and sequentially filtered with sterile cellulose membranes with pore sizes of 0.45 μm and 0.22 μm (Merck Millipore, USA). The virus-sized particles were then concentrated by ultracentrifugation at 100,000 × g and 4°C for 3 hours (Beckman Optima XL-90, SW 40Ti fixed-angle rotor). The obtained pellets were resuspended in 500 μl of Tris-HCL buffer (pH 7.5), and non-particle-protected nucleic acids were removed by digestion as described previously [21]. Briefly, 100 μl of the resuspended pellets was treated with a nuclease cocktail of DNase TURBO (32 U) (Ambion, Vilnius, Lithuania) and RNase Cocktail (2 U) (Ambion, Vilnius, Lithuania) in 40 μl of 1X TURBO DNase buffer. The reaction was incubated at 37°C for 60 minutes and then at room temperature for 5 minutes with 40 μl of DNase Inactivator. The RNA and DNA nucleic acids were purified according to Ullmann *et al*. [22] using a commercial kit (QIAamp MinElute Virus Spin, Qiagen, Hilden, Germany) following the manufacturer's recommended protocol. The RNA was then quantified using a commercial fluorometer (Qubit 2.0, Invitrogen, Carlsbad, CA, USA) with an RNA Assay kit (Invitrogen, Carlsbad, CA, USA). First-strand cDNA was then synthesized with random hexamers using the SuperScript First-Strand Synthesis System kit (Invitrogen, CA, USA) with the following mix: 1 μl of dNTP mix (10 mM), 1 μl of random hexamers (50 ng/μl), 7 μl of RNA and 10 μl of nuclease-free water. The reaction was incubated at 65°C for 5 minutes and then at 4°C for 2 minutes. A mix of 2 μl of 10X RT buffer, 4 μl of MgCl_2_ (25 mM), 2 μl of DTT (0.1 M) and 1 μl of RNaseOUT (40 U/μl) was then added, and the resulting mixture was incubated at room temperature for 2 minutes. One microliter of SuperScript III RT (200 U/μl) was then added, and the reaction was incubated at room temperature for 10 minutes, 42°C for 50 minutes and 70°C for 15 minutes. Second-strand DNA was obtained with Large Fragment of DNA Polymerase I (Klenow fragment, Invitrogen, CA, USA) with the following mix: 3 μl of 10X REact 2 buffer, 5 μl of dNTP mix (10 mM), DNA Polymerase I (2.5U), 1 μl of T4 DNA ligase, 1 μl of RNase H, cDNA (1 μg) and 30 μl of nuclease-free water. The reaction was incubated for 60 minutes at 15°C, and double-stranded DNA was then purified with commercial magnetic beads (Agencourt AMPure XP system, Beckman Coulter, USA), quantified by fluorometry using a commercial dsDNA kit (dsDNA High-Sensitive Kit, Invitrogen, Carlsbad, CA, USA) and electrophoresed in a 1% agarose gel. These procedures were performed simultaneously for each of the 12 pools.

### Library preparation and sequencing

Library preparation and sequencing were performed at the Instituto de Fisiología Celular, Unidad de Biología Molecular, UNAM. The initial evaluation of the quality and size distribution of the purified DNA was performed with the Agilent 2200 TapeStation using the Agilent Genomic DNA ScreenTape (Agilent Technologies, Santa Clara, CA, USA). The DNA libraries were then constructed using a Nextera-XT DNA Sample Preparation Kit (Illumina, San Diego CA, USA) and a Nextera XT Index Kit (Illumina) according to the manufacturer's specifications. After the fragmentation and amplification reactions, the DNA libraries were analyzed with the Agilent 2200 TapeStation using the Agilent High-Sensitivity D1000 ScreenTape and Reagents (Agilent Technologies). The 12 indexed libraries were pooled, mixed with a PhiX Control Kit v3 (Illumina) and sequenced using the MiSeq Illumina platform with a MiSeq Reagent Kit (version 3) to obtain 150-bp paired reads.

### Bioinformatics analysis

Raw reads were mapped onto the reference genome of *Anas platyrhynchos* (GenBank assembly: BGI_duck_1.0 [GCA_000355885.1]) using BWA-MEM v0.7.15 [23], and bacterial reads were removed using Kraken (Galaxy Version 1.2.3). The remaining reads were compared to a customized viral database from the NCBI (ftp://ftp.ncbi.nlm.nih.gov/genbank; gbvrl 1–50, accessed July 25^th^, 2018) using BLASTn (v2.7.1+) with an E value of 10^‒4^. The BLASTn output was analyzed and visualized using MEGAN (v6.12.3) [24] for the assignments of taxonomic families (LCA weighted = 80, minimum support = 3, minimum score = 40.0, max expected = 0.0001, top percent = 5.0, min identity = 80). Based on BLASTn output, sequences classified as viruses were then compared to a viral protein database constructed with all viral sequences from the NCBI protein database (nr, accessed August 20^th^, 2018) using BLASTx (v2.7.1+) with an E value of 10^‒5^. Furthermore, rarefaction curves and Shannon-Weaver indexes (richness) were calculated from the normalized counts (reads were normalized to the smallest sample size) at the family level using MEGAN. To compare the viral compositions, a Bray–Curtis dissimilarity matrix was constructed based on the abundances of viral families, and a principal coordinate analysis of the Bray–Curtis matrix was performed with MEGAN.

## Results

A total of 47,352,370 reads with Phred quality scores > 30 were obtained (NCBI BioProject accession no. PRJNA449682 and Sequence Read Archive accession no. SRP140672). The sequences were filtered by quality, and the host and bacteria sequences were removed. The BLASTn results and the taxonomic classification based on MEGAN are summarized in Table 1.

**Table 1:** Reads assigned to virus taxa.

Pool sample^a^	Species	Illumina reads (>30Q)	Reads removed^b^	# reads to viral taxa	Shannon-Weaver index^c^
1	*A*. *acuta*	1,807,765	310,129	77,895	1.1
2	*M*. *americana* (I)	2,412,921	109,057	43,050	3.1
3	*M*. *americana* (II)	1,101,466	134,664	57,393	3.3
4	*S*. *clypeata* (I)	1,691,373	198,205	91,099	0.9
5	*S*. *clypeata* (II)	1,586,935	118,611	87,019	1.8
6	*S*. *clypeata* (III)	2,056,522	341,458	128,390	1.0
7	*S*. *cyanoptera*	1,966,114	397,044	113,774	1.0
8	*S*. *discors*	2,467,922	288,267	158,928	3.1
9	*M*. *strepera* (I)	1,695,263	154,071	95,543	3.4
10	*M*. *strepera* (II)	1,745,842	216,957	210,070	3.7
11	*O*. *jamaiciencis* (I)^d^	1,762,231	111,142	30,371	3.3
12	*O*. *jamaiciencis* (II)^d^	2,051,149	83,249	21,835	3.6

The results of a BLASTn search of reads classified to virus taxa using the GenBank virus database are shown.

The number in parentheses represents a different pool of the same species with different individuals.

^a^ Each pool includes five individuals of the same species.

^b^ Reads from host genome and bacteria.

^c^ Indexes calculated at family level using normalized reads.

^d^ Diving duck species.

**Fig 1:**
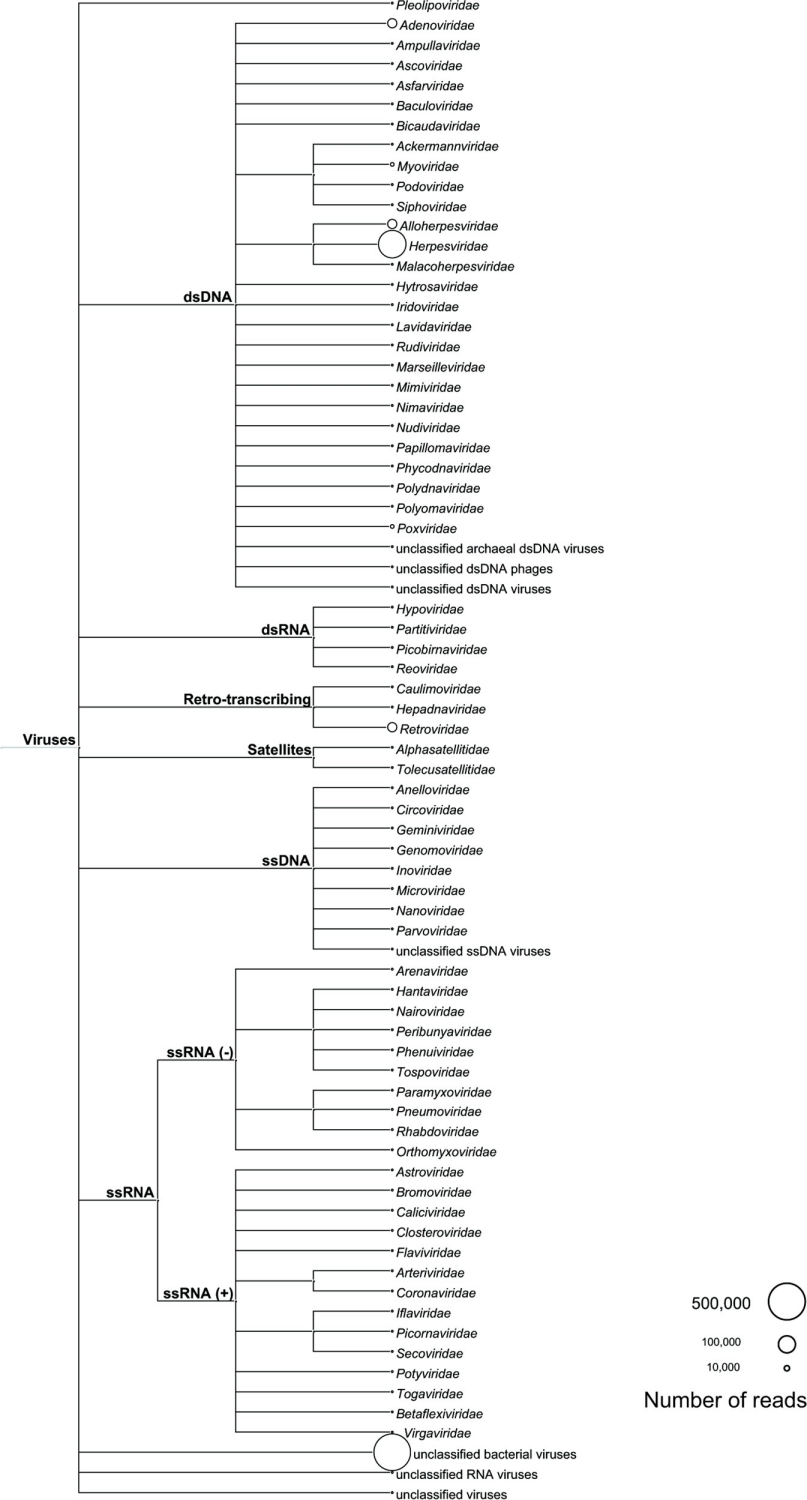
Overall abundances of viral families identified in wild ducks. A lowest common ancestor (LCA) tree was constructed based on the data obtained with a BLASTn search. The results were analyzed and visualized using MEGAN. The sizes of the circles correspond to the abundances of the total reads in the 12 sample pools.

Overall, the *Herpesviridae*, *Alloherpesviridae*, *Adenoviridae*, *Retroviridae* and *Myoviridae* families showed the highest abundances in the duck species (Fig 1). DNA and RNA viral sequences represented 95% and 5% of the viral genetic material, respectively, and eukaryotic-related viral sequences and phages represented 59% and 41% of the total material, respectively.

The virus species identified by BLASTx of the top viral families in the samples of wild duck feces are shown in Table 2. Viral sequences related to birds, humans, bacteria and fish were found. Additionally, more than 146 vertebrate-, insect-, bacteria- and plant-related virus species were also identified (S1 Table).

**Table 2:** Virus species of the top families identified in feces of wild ducks.

Family	Species
*Myoviridae*	*Escherichia virus P1*
*Alloherpesviridae*	*Cyprinid herpesvirus 1*
*Herpesviridae*	*Columbid alphaherpesvirus 1*
	*Human betaherpesvirus 5*
*Retroviridae*	*Avian leukosis virus*
	*Reticuloendotheliosis virus*

Sequences classified as viruses were compared to a viral protein database using BLASTx.

**Fig 2:**
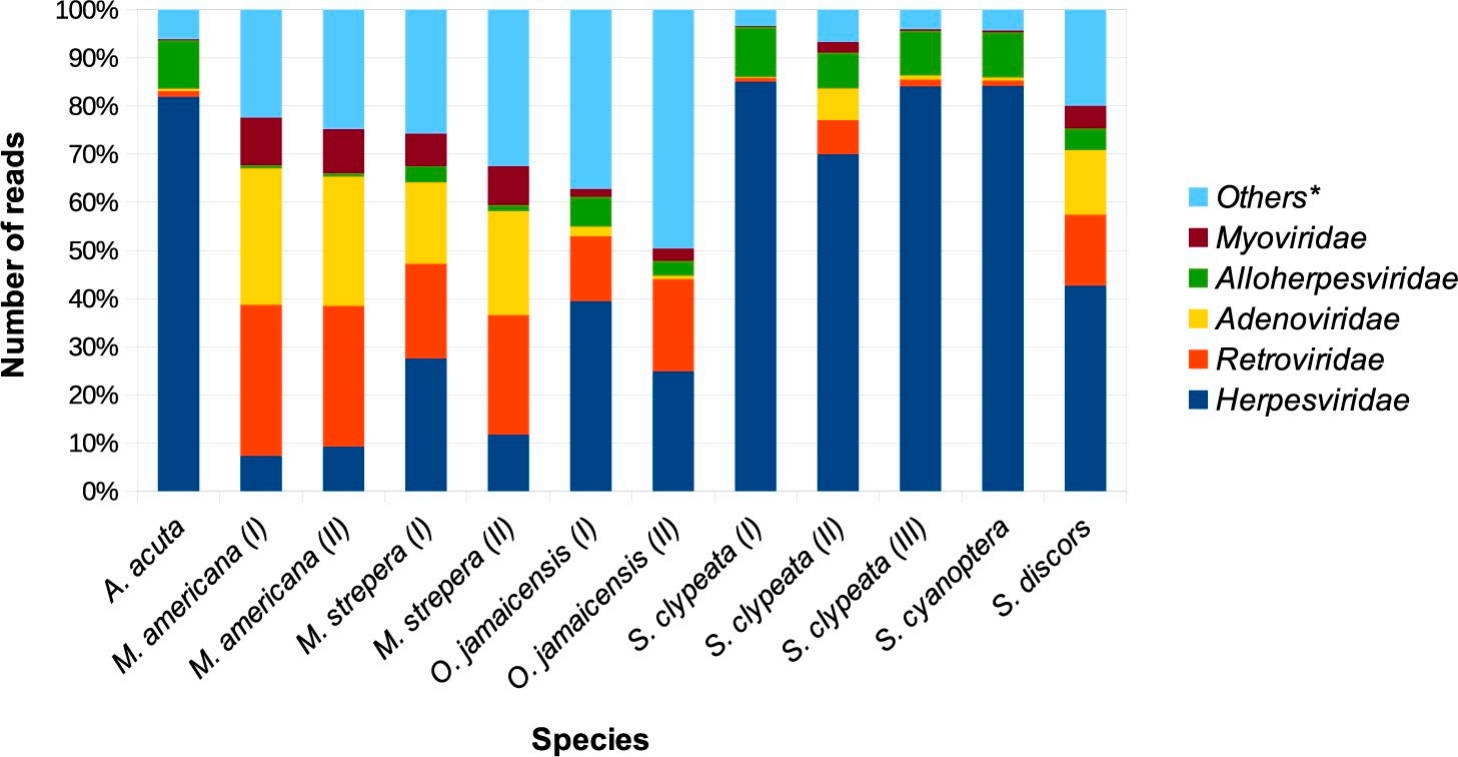
Comparison of top viral families identified in the species of wild ducks. The reads from the BLASTn search were normalized and analyzed with MEGAN. *The term “Others” represents the rest of the identified viral families (63) that were not shown in the figure and had an abundance < 1%.

The rarefaction curves of all the samples trended to a horizontal asymptote (S1 Fig), which indicated that most of the viral families had been measured. The estimated richness among the species was heterogeneous: the lowest number of 0.9 was obtained for the species *S*. *clypeata*, and the highest number of 3.7 for the species *M*. *strepera*. Samples of the same species had a similar viral richness. The comparisons of the reads assigned to viral families in the different species of wild ducks are represented in Fig 2. The relative abundances of the main identified viral families (*Herpesviridae*, *Adenoviridae*, *Retroviridae*, *Alloherpesviridae* and *Myoviridae*) showed differences among the species of ducks. The most strongly represented family in the group of samples was *Herpesviridae*, which had an abundance of up to 85%. However, the percentage ranged from ~7 to 85%. The *Retroviridae* and *Adenoviridae* families had an abundance of 1–31% and ~0.5–28% respectively. The percentage from the *Myoviridae* family in the species *M*. *americana* and *M*. *strepera* ranged from ~7% to 10%, and in the species *S*. *clypeata*, *S*. *cyanoptera*, *S*. *discors*, *A*. *acuta* and *O*. *jamaicensis* was <5%. The abundance of the *Alloherpesviridae* family was from 1 to 11%, and the rest of viral families that represented <1% were ranged between 5 to 50%.

**Fig 3:**
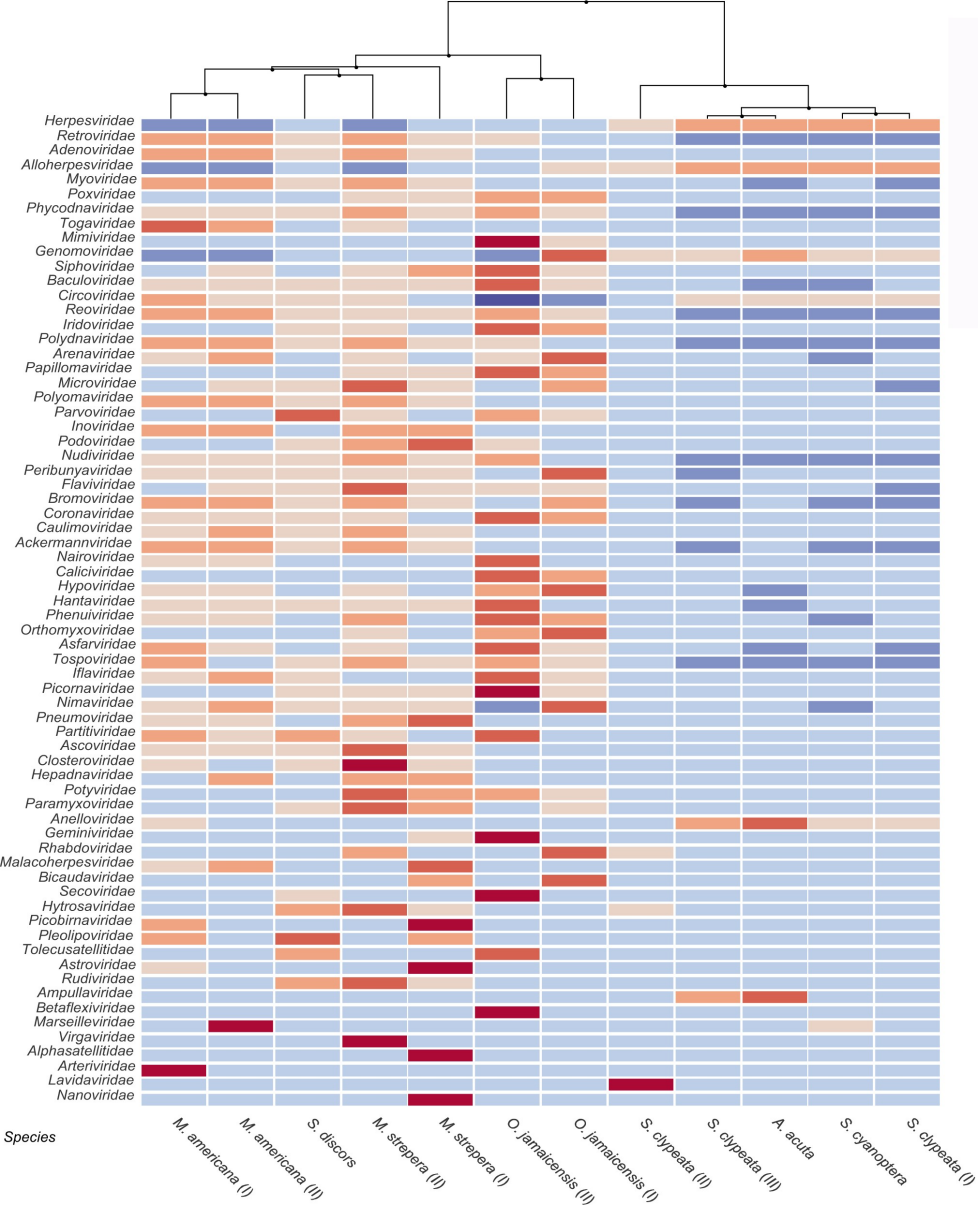
Heatmap displaying the relative abundances of the viral families. The relative abundances of the viral families found in wild ducks according to the BLASTn matches are shown. The reads were normalized, analyzed and visualized with MEGAN. The color coding indicates the abundances relative to the mean (red shows high abundance, and blue indicates low abundance).

A heatmap of the relative abundances of all the viral families identified in the different species of ducks is presented in Fig 3. The duck species were grouped into two clades in the dendrogram according to similarities in the abundances of the identified viral families. The pools grouped in the right clade of the dendrogram exhibited lower abundances (blue) in most families. The duck species *A*. *acuta* and *S*. *clypeata* (III) shared a similar composition with low abundances of most of the viral families, and the samples of the species *S*. *clypeata* (I) and *S*. *cyanoptera*, also exhibited a similar composition. The pool of *S*. *clypeata* (II) was grouped into a different subclade from the other species. In the left clade, the pools of *O*. *jamaicensis* (diving ducks) were grouped into a different subclade from the left clade species. The *O*. *jamaicensis* species had greater abundances (red) of most families compared with the other species. In two pools of *M*. *americana*, very similar compositions were observed, and the abundances of most of the viral families were very homogeneous, i.e., no particular family was dominant. Similar composition was observed in the pools of *S*. *discors* and *M*. *strepera* (II), but a different abundance was found in pool I of *M*. *strepera*, which resulted into it being grouped into a different subclade.

**Fig 4:**
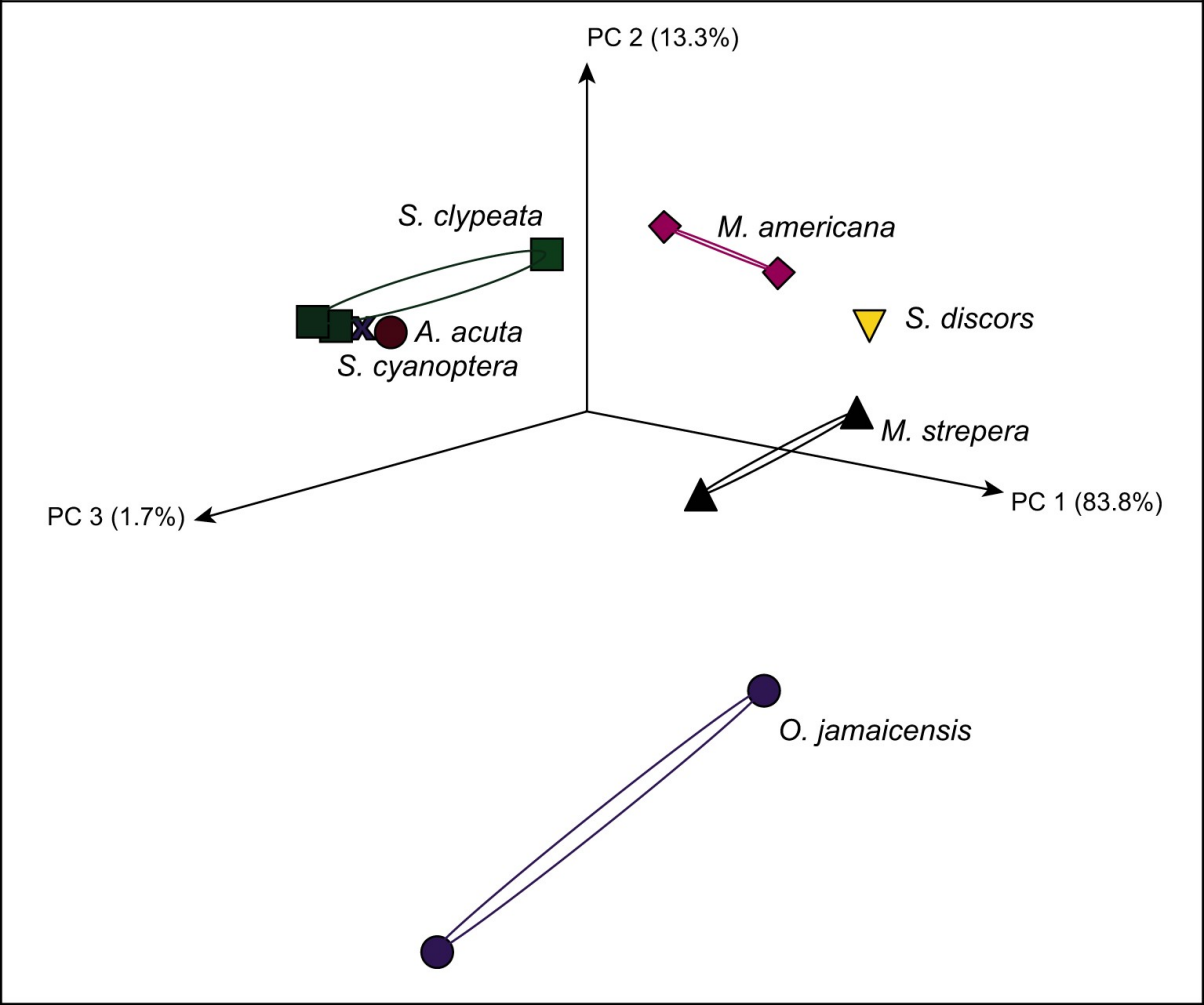
Principal coordinate analysis (PCoA) of the virome compositions of wild ducks. The analysis was based on a Bray–Curtis dissimilarity matrix that was constructed with MEGAN using normalized reads. The duck species are represented by color figures. The variances explained by the PCs are indicated in parentheses on the axes.

A principal coordinate analysis was performed to visualize the clustering patterns of the samples. This analysis revealed that the virome compositions were clustered according to the species of wild ducks. As illustrated in Fig 4, 83% of the variation observed was in PC 1, and the pools of *M*. *americana*, *M*. *strepera* and *S*. *discors* exhibited similar patterns of diversity and were grouped into clusters. The species *A*. *acuta* and *S*. *cyanoptera* had a slightly similar clustering pattern and were grouped closed to the species *S*. *clypeata*. The pools of the species *O*. *jamaicensis* were clustered far from other species.

## Discussion

The results obtained in this study extend the knowledge of the intestinal viromes in different species of migratory wild ducks that share the same natural wetland during their winter stayover. The overall samples presented a greater abundance of DNA viruses than RNA viruses and a higher proportion of eukaryotic viruses than phages. The proportions of DNA compared with RNA viruses were different from those obtained in a previous study of domestic waterfowl. Zhao *et al*. [25] found a higher percentage of RNA and eukaryotic viruses in the feces of domestic ducks, whereas Fawaz *et al*. [26] reported a higher proportion of phages and DNA viruses in cloaca samples from domestic ducks.

The fecal analysis of wild ducks revealed a complex diversity of vertebrate-, insect-, bacteria- and plant-related viruses. In fact, sequences belonging to *Herpesviridae* family and species such as *Columbid alphaherpesvirus 1* which are associated with disease in pigeons, owls and falcons, were found [27, 28]. Moreover, *Human betaherpesvirus 5* were also identified, this is a ubiquitous herpesvirus that cause generally asymptomatic or self-limiting infections in healthy adult hosts and congenital infection in newborn infants [29]. The *Herpesviridae* family has been previously detected in fresh water samples [30] and in the fecal virome of species such as bats [31, 32], wild rodents [33] and domestic ducks [25, 26]. However, no virus species belonging to this family has been previously reported in virome of ducks, and the abundance of this family in domestic ducks was lower than that obtained in this study. We identified six phage families, namely, *Myoviridae*, *Siphoviridae*, *Podoviridae*, *Ackermannviridae*, *Inoviridae* and *Microviridae*, as well as sequences related to unclassified bacterial viruses (41%). These families have been previously reported in domestic ducks, but the percentages of phages found in free-living and farm ducks ranged from 1 to 77% [25, 26]. In this study, *Adenoviridae* viruses were detected in all examined species of wild ducks, however, no virus species could be identified by BLASTx. This family has been detected in the intestinal virome, as determined through the deep sequencing of samples of domestic ducks [25], broiler chickens [34] and bats [35, 36]. The virus species identified from the *Retroviridae* family are associated with infectious diseases in poultry. The *Avian leukosis virus* and others leukosis/sarcoma viruses induce a wide range of neoplastic conditions is widespread and cause significant economic losses [37, 38]. The *Reticuloendoteliosis virus* infection is common in flocks of chickens, turkeys and ducks, and induce chronic lymphomas and an immunosuppressive runting disease [39]. Additionally, these viruses have been reported in wild ducks [40–42]. We identified viruses related to the *Alloherpesviridae* family, and within this family, virus species of the *Cyprinivirus* genus are associated with hemorrhagic diseases in fish [43, 44]. Fish-related viruses have been previously observed in domestic ducks [26], but the previous study did not mention families or species. Moreover, we identified 22 families that had not been previously detected in fecal samples of migratory wild ducks and 36 viral families that had been previously observed in other studies of fecal or cloacal samples of ducks [25, 26, 45]. Therefore, viruses relevant to veterinary medicine that are possibly disseminated by wild ducks should be monitored, and the possible role of wild ducks harboring human viruses should be further studied. The presence of these vertebrate-related viruses in clinically healthy ducks is not fully understood: it is possible that these duck-associated viruses are persistently infecting ducks. On the other hand, ducks might be carrying viruses present in environments impacted by human activities such as livestock, poultry, fishing, and wastewater. Additionally, the prokaryotic-related viruses might infect bacteria present in the intestine of ducks or in the aquatic ecosystem.

Although the main viral families were detected in all the different analyzed species of ducks, the heatmap analysis showed differences in the abundances of viral families between species and between samples of same species of wild ducks. The identification of similarities in the intestinal communities of the viruses in ducks that share an ecosystem was expected because it has been suggested that the environment influences the virome compositions in individuals who share habitats [46]. The differences in viral abundance could be explained by factors such as age, diet and individual variations. The results revealed that *O*. *jamaicensis* was the species with the highest abundance of viral families and exhibited high richness indexes. These ducks are diving species and feed on aquatic organisms, seeds and vegetation from the bottom of the water, whereas the others species investigated in this study are dabbling ducks that typically feed on the surface or in the shallows, although they can also graze in fields [47]. The foraging habits of the diving and dabbler ducks could explain the differences in the intestinal virome profiles. Previous studies have not demonstrated that diet influences the composition of the virome in wild birds, but there is evidence suggesting that feeding behavior shapes the structure of the virome in bat species [32]. Moreover, we found variations in the virome profile among species, and this feature has been documented in three different bat species [48]. Therefore, differences in the viral communities of feces from wild ducks can be attributed to their specific ecological niches and to their diets.

Coordinate analysis exhibited similar patterns in virome structure, but also differences between species of wild ducks. Previous studies have indicated the existence of inter-personal variability in the virome composition of humans and domestic ducks [25, 49]. This variability could be explained by multiple factors, such as differences in the ages of the animals or in the levels of immune responses. In chickens, humans and pigs, the intestinal viral community changes according to age [8, 9]. Although variations in viral abundance that are linked to age have not been demonstrated in wild ducks, information regarding the susceptibility of younger mallards to influenza virus A is available, and this infection is the result of an immature immune system [16]. We also need to consider the possible interactions between viruses as a factor that can alter the immune response or the infectiousness of a pathogen that is not related to the intestinal community (i.e., heterologous immunity) [50]. Previous studies have not demonstrated whether infection with some virus could alter the virome structure, but it has been shown that individual infection with a virus can enhance the immune response against a different virus in experimentally infected healthy mice [51]. Moreover, Ganz et al. [52] suggested that infection with a specific virus could alter the bacteriome in wild ducks. Therefore, in the future, we need to continue studying the interactions between viruses and the virome in the intestinal communities of clinically healthy animals and how these interactions allow outbreaks of infectious diseases in domestic animals, and we should also explore the biotic and abiotic factors that influence the variations in relative abundances between different species of duck.

## Conclusions

The relative abundance of duck-related viruses in different species of wild ducks sharing the same habitat is diverse. The role of new viruses that might be found in the intestine of wild ducks is not fully understood, and we hypothesize that these viruses likely inhabit the environment and that their occurrence depends on certain conditions in each ecosystem, such as anthropogenic impact and agricultural and livestock activity. Further study of the viruses harbored by wild migratory ducks is important for monitoring the potential zoonotic risk or outbreaks in domestic animals. Additionally, the factors that determine the compositions of the viromes of different species of wild ducks and their influence in farmed ducks should be elucidated.

## Supporting information

S1 Table Virus species identified in feces of wild ducks. Sequences classified as viruses were compared to a viral protein database using BLASTx.
(DOCX)

S1 Fig Rarefaction curves for 12 fecal samples. Rarefaction curves from the BLASTn search were created with MEGAN at the family level.
(TIF)
