# Supplementary material for: Fecal virome composition of migratory wild duck species
Source: PLoS One. 2018 Nov 21;13(11):e0206970. doi: 10.1371/journal.pone.0206970 (PMC6248937; doi:10.1371/journal.pone.0206970)
Supplement: S1 Table — Sequences classified as viruses were compared to a viral protein database using BLASTx. (DOCX) [file pone.0206970.s001.docx]

**S1 Table. Virus species identified in feces of wild ducks. S**equences classified as viruses were compared to a viral protein database using BLASTx.

| ***Ampullaviridae*** |
| --- |
| *Acidianus bottle-shaped virus* |
| ***Baculoviridae*** |
| *Autographa californica multiple nucleopolyhedrovirus* |
| ***Myoviridae*** |
| *Escherichia virus P1* |
| *Salmonella phage SJ46* |
| *Ralstonia phage RSY1* |
| *Staphylococcus virus Remus* |
| *Synechococcus phage ACG-2014c* |
| *Synechococcus phage S-PM2* |
| *Bacillus virus G* |
| *Acinetobacter phage YMC13/03/R2096* |
| *Bacillus phage BM5* |
| *Bdellovibrio phage phi1422* |
| *Cellulophaga phage phiSM* |
| *Clostridium phage c-st* |
| *Cronobacter phage vB_CsaM_GAP32* |
| *Mycobacterium phage Myrna* |
| *Prochlorococcus phage P-TIM68* |
| *Pseudomonas phage PPpW-3* |
| *Rhizobium phage RHE* |
| *Salmonella phage SEN34* |
| *Shigella phage SfIV* |
| *Synechococcus phage Syn19* |
| ***Podoviridae*** |
| *Pseudomonas virus F116* |
| *Dinoroseobacter phage DFL12phi1* |
| *Salmonella virus HK620* |
| *Enterobacteria phage VT2phi_272* |
| *Cellulophaga phage phi14:2* |
| *Ralstonia phage RSK1* |
| *Rhodoferax phage P26218* |
| *Xylella phage Xfas53* |
| ***Siphoviridae*** |
| *Bacillus virus Riggi* |
| *Burkholderia virus phi1026b* |
| *Burkholderia virus phiE125* |
| *Bacillus phage SPO2* |
| *Enterobacteria phage phi80* |
| *Mycobacterium virus Mosmoris* |
| *Arthrobacter virus Mudcat* |
| *Propionibacterium virus P1001* |
| *Propionibacterium virus PHL116M00* |
| *Staphylococcus virus 37* |
| *Staphylococcus phage StB12* |
| *Streptococcus virus 7201* |
| *Gordonia virus OneUp* |
| *Bacillus virus SPbeta* |
| *Staphylococcus phage SPbeta-like* |
| *Acinetobacter phage vB_AbaS_TRS1* |
| *Actinoplanes phage phiAsp2* |
| *Bacillus phage PBC1* |
| *Bacteroides phage B40-8* |
| *Brevibacillus phage Sundance* |
| *Burkholderia phage Bcep176* |
| *Clostridium phage phiCD111* |
| *Clostridium phage phiCTP1* |
| *Enterococcus phage phiEf11* |
| *Enterococcus phage vB_EfaS_IME197* |
| *Geobacillus virus E3* |
| *Helicobacter phage phiHP33* |
| *Idiomarinaceae phage Phi1M2-2* |
| *Klebsiella phage phiKO2* |
| *Lactobacillus phage Lrm1* |
| *Lactococcus phage TP901-1* |
| *Microbacterium phage Min1* |
| *Mycobacterium phage Dori* |
| *Paracoccus phage vB_PmaS_IMEP1* |
| *Pseudomonas phage MD8* |
| *Pseudomonas phage PAJU2* |
| *Pseudomonas phage YMC11/02/R656* |
| *Pseudomonas phage YMC11/07/P54_PAE_BP* |
| *Rhizobium phage 16-3* |
| *Staphylococcus phage phiRS7* |
| *Staphylococcus phage StB20-like* |
| *Streptococcus phage phiNJ2* |
| *Synechococcus phage S-SKS1* |
| *Paenibacillus virus Vegas* |
| ***Alloherpesviridae*** |
| *Cyprinid herpesvirus 1* |
| ***Herpesviridae*** |
| *Columbid alphaherpesvirus 1* |
| *Human alphaherpesvirus 1* |
| *Cercopithecine alphaherpesvirus 9* |
| *Cercopithecine betaherpesvirus 5* |
| *Human betaherpesvirus 5* |
| *Cynomolgus cytomegalovirus* |
| *Elephantid betaherpesvirus 1* |
| *Bovine gammaherpesvirus 4* |
| ***Iridoviridae*** |
| *Red seabream iridovirus* |
| *European catfish virus* |
| ***Mimiviridae*** |
| *Cafeteria roenbergensis virus* |
| *Acanthamoeba polyphaga mimivirus* |
| *Megavirus chiliensis* |
| ***Phycodnaviridae*** |
| *Only Syngen Nebraska virus 5* |
| *Phaeocystis globosa virus* |
| *Aureococcus anophagefferens virus* |
| *Chrysochromulina ericina virus* |
| ***Polydnaviridae*** |
| *Cotesia congregata bracovirus* |
| ***Polyomaviridae*** |
| *Macaca mulatta polyomavirus 1* |
| ***Poxviridae*** |
| *Canarypox virus* |
| *Molluscum contagiosum virus* |
| *BeAn 58058 virus* |
| ***Caulimoviridae*** |
| *Mulberry badnavirus 1* |
| *Sugarcane bacilliform IM virus* |
| *Cycad leaf necrosis virus* |
| *Strawberry vein banding virus* |
| ***Retroviridae*** |
| *Avian carcinoma Mill Hill virus 2* |
| *Avian leukosis virus* |
| *Avian sarcoma virus CT10* |
| *Fujinami sarcoma virus* |
| *Rous sarcoma virus* |
| *Avian musculoaponeurotic fibrosarcoma virus AS42* |
| *Avian sarcoma virus* |
| *UR2 sarcoma virus* |
| *Y73 sarcoma virus* |
| *Jaagsiekte sheep retrovirus* |
| *Squirrel monkey retrovirus* |
| *Bovine retrovirus CH15* |
| *Walleye dermal sarcoma virus* |
| *Feline leukemia virus* |
| *Hardy-Zuckerman feline sarcoma virus* |
| *Koala retrovirus* |
| *Moloney murine sarcoma virus* |
| *Murine leukemia virus* |
| *Reticuloendotheliosis virus* |
| *Abelson murine leukemia virus* |
| *Baboon endogenous virus* |
| *RD114 retrovirus* |
| *Woolly monkey sarcoma virus* |
| *Avian endogenous retrovirus EAV-HP* |
| *Human endogenous retrovirus K* |
| ***Circoviridae*** |
| Not identified species |
| ***Genomoviridae*** |
| *Caribou associated gemykrogvirus 1* |
| *Bemisia-associated genomovirus AdO* |
| ***Inoviridae*** |
| *Ralstonia phage p12J* |
| *Stenotrophomonas phage phiSHP2* |
| ***Microviridae*** |
| *Enterobacteria phage phiX174 sensu lato* |
| *Bdellovibrio virus MH2K* |
| *Gokushovirinae Bog1183_53* |
| *Gokushovirinae Fen672_31* |
| *Gokushovirinae GAIR4* |
| ***Parvoviridae*** |
| *Dipteran ambidensovirus 1* |
| *Parus major densovirus* |
| *Adeno-associated dependoparvovirus A* |
| *Adeno-associated dependoparvovirus B* |
| *Avian dependoparvovirus 1* |
| *Bearded dragon parvovirus* |
| *Rodent protoparvovirus 1* |
| *Ungulate protoparvovirus 1* |
| ***Orthomyxoviridae*** |
| *Influenza A virus* |
| ***Flaviviridae*** |
| *Hepacivirus C* |
| **Unclassified viruses** |
| *Brucella phage BiPBO1* |
| *Phage Gifsy-1* |
| *Propionibacterium phage PFR2* |
| *Streptococcus phage 20617* |
| *Shahe heteroptera virus 4* |
| *Lake Sarah-associated circular virus-36* |
| *Lake Sarah-associated circular virus-50* |
| *Rhizobium phage RHEph10* |
| *Idiomarinaceae phage 1N2-2* |
| *Marinomonas phage P12026* |
| *Salicola phage CGphi29* |
| *Unclassified viruses* |
| *Pandoravirus dulcis* |
| *Pandoravirus salinus* |
| *Tetraselmis viridis virus S20* |
