# Supplementary figures and images for: Fecal virome composition of migratory wild duck species
Source: PLoS One. 2018 Nov 21;13(11):e0206970. doi: 10.1371/journal.pone.0206970 (PMC6248937; doi:10.1371/journal.pone.0206970)

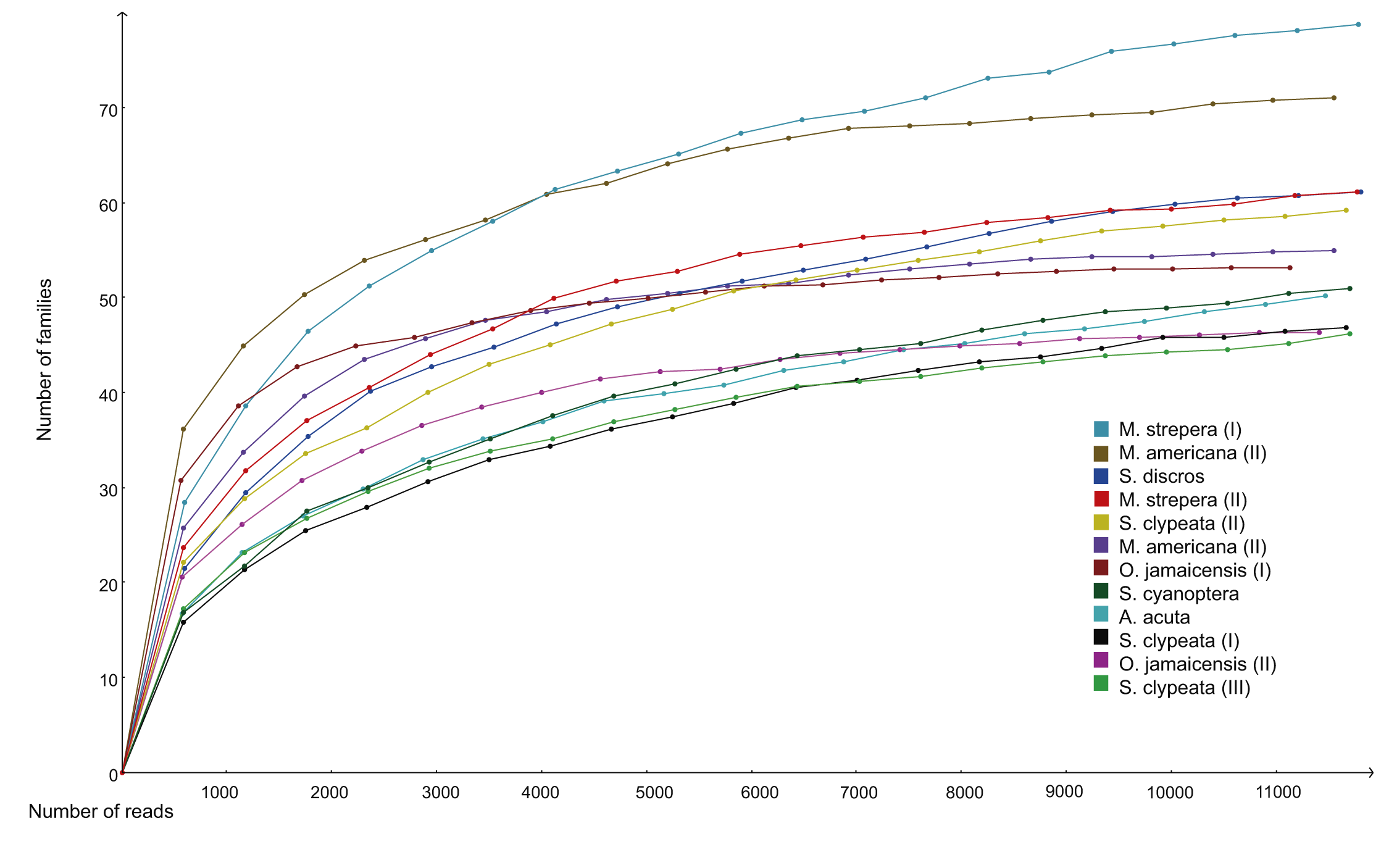

Supplement: S1 Fig — Rarefaction curves from the BLASTn search were created with MEGAN at the family level. (TIF) [file pone.0206970.s002.tif]
